# Ultrahigh Electrostrictive Effect in Lead-Free Sodium Bismuth Titanate-Based Relaxor Ferroelectric Thick Film

**DOI:** 10.3390/nano14171411

**Published:** 2024-08-29

**Authors:** Yizhuo Li, Jinyan Zhao, Zhe Wang, Kun Zheng, Jie Zhang, Chuying Chen, Lingyan Wang, Genshui Wang, Xin Li, Yulong Zhao, Gang Niu, Wei Ren

**Affiliations:** 1 State Key Laboratory for Manufacturing Systems Engineering, Electronic Materials Research Laboratory, Key Laboratory of the Ministry of Education, School of Electronic Science and Engineering, Xi’an Jiaotong University, Xi’an 710049, Chinawren@xjtu.edu.cn (W.R.); 2 Laboratory of Sensitive Materials and Devices, Shandong Department of Education, School of Materials Science and Engineering, Liaocheng University, Liaocheng 252059, China; 3 Shanghai Institute of Ceramics, Chinese Academy of Sciences, Shanghai 200050, China; genshuiwang@mail.sic.ac.cn (G.W.);; 4 State Key Laboratory for Manufacturing Systems Engineering, School of Mechanical Engineering, Xi’an Jiaotong University, Xi’an 710049, China; 5 The International Joint Laboratory for Micro/Nano Manufacturing and Measurement Technology, Xi’an Jiaotong University, Xi’an 710049, China

**Keywords:** sodium bismuth titanate, electrostrictive effect, lead-free, film, relaxor, ferroelectrics

## Abstract

In recent years, the development of environmentally friendly, lead-free ferroelectric films with prominent electrostrictive effects have been a key area of focus due to their potential applications in micro-actuators, sensors, and transducers for advanced microelectromechanical systems (MEMS). This work investigated the enhanced electrostrictive effect in lead-free sodium bismuth titanate-based relaxor ferroelectric films. The films, composed of (Bi_0.5_Na_0.5_)_0.8−x_Ba_x_Sr_0.2_TiO_3_ (BNBST, x = 0.02, 0.06, and 0.11), with thickness around 1 μm, were prepared using a sol-gel method on Pt/TiO_2_/SiO_2_/Si substrates. By varying the Ba^2+^ content, the crystal structure, morphology, and electrical properties, including dielectric, ferroelectric, strain, and electromechanical performance, were investigated. The films exhibited a single pseudocubic structure without preferred orientation. A remarkable strain response (*S* > 0.24%) was obtained in the films (x = 0.02, 0.06) with the coexistence of nonergodic and ergodic relaxor phases. Further, in the x = 0.11 thick films with an ergodic relaxor state, an ultrahigh electrostrictive coefficient *Q* of 0.32 m^4^/C^2^ was achieved. These findings highlight the potential of BNBST films as high-performance, environmentally friendly electrostrictive films for advanced microelectromechanical systems (MEMS) and electronic devices.

## 1. Introduction

With the rapid development of microelectromechanical systems (MEMS) and other high-precision electronic devices, the demand for materials possessing strong and controllable electromechanical coupling has significantly increased [1,2]. Ferroelectric films, which play an important role in the development of functional devices, are particularly promising, thanks to their electromechanical conversion ability. An electric field-induced strain, which converts electricity into mechanical energy, can be described as [3]

(1) $$S=S_{1}+S_{2}+\ldots =dE+ME^{2}+\ldots$$

where *S*_1_ represents the first-order piezoelectric effect, and *S*_2_ represents the second-order electrostrictive effect, respectively; *d* is the piezoelectric coefficient, *M* is the electrostrictive coefficient, and *E* is the applied electric field. The strain related to the higher-order effects are typically negligible in ferroelectric materials.

The piezoelectric effect can only be obtained after the materials are poled and their operational temperature is limited by Curie or depoling temperature. Electrostriction, present in all dielectric materials, has the advantages of high actuation efficiency, low hysteresis, and excellent stability under high-temperature and high-frequency operation [4,5,6,7]. These properties make ferroelectric films with high electrostrictive effects ideal candidates for applications in actuators and transducers [8]. The electrostriction-related strain, *S*_2_, involves a quadratic dependence on the polarization [9], which is mathematically represented as follows [4,10,11]:

(2) $$S_{2}=Q{\left(\varepsilon_{r}E\right)}^{2}=Q\cdot P^{2}$$

where *Q* is the electrostrictive coefficients, *ε_r_* is the dielectric constant, *E* is the applied electric field, and *P* is the polarization. Traditional lead-containing materials, such as lead magnesium niobate (PMN), often with small amounts (<15 mol%) of lead titanate (PMN-PT), i.e., relaxor ferroelectric materials, occupy the electrostrictive actuator applications due to their high strain [12,13,14,15]. An electrostrictive coefficient of 0.035 m^4^/C^2^ was reported in the Sm^3+^ doped PMN-PT ceramics [16]. The potential electrostrictive effect was also reported in lead-based thin films. The electrostrictive coefficients of 0.05 m^4^/C^2^ and 0.082 m^4^/C^2^ were obtained in PbTiO_3_ ferroelectric films [17] and PbZrO_3_ antiferroelectric films [18], respectively. However, in recent years, researchers have focused on developing environmentally friendly, lead-free electrostrictive materials in consideration of environmental protection and sustainable development. *Q* values exceeding 0.04 m^4^/C^2^ are generally reported in lead-free Ba(Zr_0.2_Ti_0.8_)O_3_-(Ba_0.7_Ca_0.3_)TiO_3_ (BCZT) system ceramics [19]. A *Q* value of 0.03 m^4^/C^2^ was obtained in (K_0.5_Na_0.5_)NbO_3_-BaZrO_3_ (KNN based) ceramics [20]. A competitive *Q* value of 0.046 m^4^/C^2^ was achieved in Bi_0.5_Na_0.5_TiO_3_ (BNT)-based solid solutions with coexisting phases [1]. BNT-based lead-free ferroelectric materials have garnered significant attention [21,22,23,24,25]. However, current research mainly focuses on BNT-based ceramics. The investigation of the electrostrictive properties of BNT-based thin films was efficient and was characterized on the local scale by scanning probe microscopy [26,27,28]. However, macroscale performances, which are more important for film applications in devices, are still lacking. The performance of films is affected by many factors, such as annealing conditions [29,30] and film thickness [31,32], making the acquisition of high-performance films and the design of devices based on ferroelectric films challenging. Therefore, exploring high-quality BNT-based films with suitable thickness to achieve outstanding electromechanical coupling performance is an urgent task for lead-free electrostrictive actuator applications. 

A ternary solid solution, (0.8−x)(Na_0.5_Bi_0.5_)TiO_3_-0.2SrTiO_3_-xBaTiO_3_ (NBT-ST-BT) in ceramics was reported in a structural transformation from pseudocubic to tetragonal when Ba^2+^ content increased [33]. A relaxor behavior varied with the Ba^2+^ content, along with a variation of polarization and electrostrain [34]. In order to explore the electrostrictive effect in BNT-based films, (Bi_0.5_Na_0.5_)_0.8−x_Ba_x_Sr_0.2_TiO_3_ (BNBST) was prepared and investigated. The film compositions x = 0.02, 0.06, 0.11 were determined in this work because of their potential ergodic relaxor behavior with higher Ba^2+^ content. The films with designed compositions were prepared on Pt/TiO_2_/SiO_2_/Si substrate using the sol-gel method. The crystal structure, morphology, and electrical properties were investigated in detail. By varying the barium content x, we attempted to optimize the electrostrictive response and understand the underlying mechanisms driving this enhancement. This work gives insight into the development of high-performance, environmentally friendly electrostrictive films for next-generation MEMS and other advanced electronic devices.

## 2. Experimental

The BNBST films were fabricated using the chemical solution deposition (CSD) method. Initially, acetate, including sodium acetate, strontium acetate, bismuth acetate, and barium acetate, was dissolved in a mixture of ethylene glycol monomethyl ether and acetic acid, which served as solvents. To account for the potential volatilization of sodium and bismuth during the high-temperature annealing process, an excess of 10 mol% sodium acetate and 2 mol% bismuth acetate were included. Subsequently, tetrabutyl titanate was added to the mixture, and acetylacetone was included as a stabilizing agent. The prepared solution was stirred continuously at 80 °C for one hour, forming a homogeneous BNBST precursor solution. The concentration of the solution was adjusted to 0.4 mol/L by modifying the solvent content. Additionally, polyvinylpyrrolidone (PVP) was introduced to enhance the viscosity of the solution, facilitating a uniform coating process.

**Figure 1:**
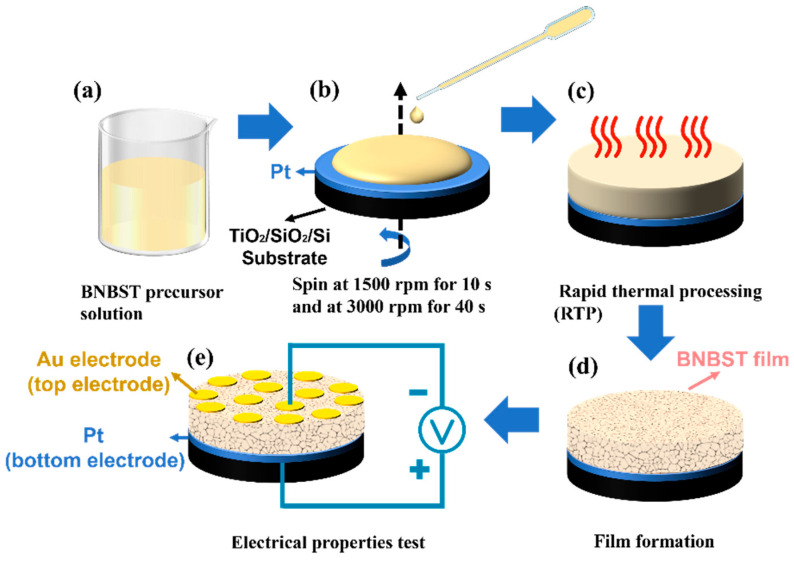
Schematic diagram of the film preparation process: (**a**) BNBST precursor solution; (**b**) spin coating; (**c**) rapid thermal processing; (**d**) film cooling and forming; (**e**) electrical properties measurement.

The following preparation process was shown in Figure 1. The deposition of the BNBST films was carried out on pretreated Pt/TiO_2_/SiO_2_/Si substrates using a spin coating technique. The precursor solution was dropped onto the substrate, which was then spun at 1500 rpm (rotation per min) for 10 s and subsequently at 3000 rpm for 40 s to ensure a uniform coating of the solution over the substrate surface. After the film deposition, the wet film underwent rapid thermal processing (RTP) heat treatment. This process included an initial pyrolysis step at 150 °C for 3 min to remove the solvent, followed by a secondary pyrolysis at 410 °C for 10 min to eliminate organic components. The crystallization of the films was achieved through annealing at 725 °C for 3 min. The films were then naturally cooled to room temperature, completing the formation of a single layer. To achieve the desired film thickness, the deposition and heating steps were repeated six times. Gold electrodes with a diameter of 0.3 mm were subsequently deposited on the surface of the films through direct current (DC) sputtering for electrical measurements.

The crystal structure of the BNBST films was analyzed using high-resolution X-ray diffraction (XRD, Empyrean, Panalytical, Almelo, The Netherlands) with a Cu Kα1 radiation source (λ = 1.5406 Å) on an Empyrean diffractometer from Panalytical (Malvern, UK). Surface and cross-sectional morphologies of the films were examined utilizing a field emission scanning electron microscope (SEM, 250 FEG, FEI Quanta, Kyoto, Japan), providing detailed images of the films’ structural features. An atomic force microscopy (AFM, Dimension Icon, Bruker, Billerica, MA, USA) was used to characterize the topography of the films. Dielectric properties of the films were measured with a precision impedance analyzer (E4980a, Agilent Technologies Inc., Santa Clara, CA, USA), which collected data across varying frequencies, DC voltages, and temperatures. Ferroelectric hysteresis loops were recorded at room temperature using a ferroelectric analyzer system (TF 2000, aixACCT GmbH, Aachen, Germany), allowing the assessment of polarization–electric field behavior. The strain behavior under electric fields was investigated using a ferroelectric analyzer equipped with a double-beam laser interferometer (aixDBLI, aixACCT GmbH, Germany). Measurements of bipolar strain curves and unipolar strain curves were conducted at room temperature under an applied electric field of 600 kV/cm, providing insights into the electrostrictive properties of the films.

## 3. Results and Discussion

**Figure 2:**
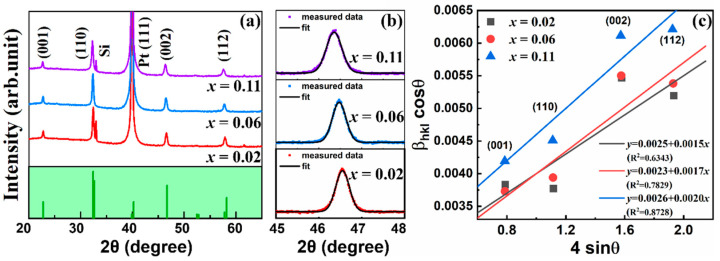
X-ray diffraction (XRD) patterns of (Bi_0.5_Na_0.5_)_0.8−x_Ba_x_Sr_0.2_TiO_3_ (x = 0.02, 0.06, 0.11) films: (**a**) wide scanning range 20°~60°; (**b**) slow scanning of (002) peak in the 45°~48° region and corresponding fit results; (**c**) the Williamson-Hall analysis plot, the dots are the data refined and calculated from Jade software, the lines are linear fitted results using modified Williamson-hall method.

The X-ray diffraction (XRD) patterns of the lead-free BNBST films were examined to investigate their crystal structures. Figure 2 illustrated the XRD patterns of the BNBST films, where 2*θ* scans from 20° to 65°. The standard BNT (PDF#89-3109) patterns were displayed for reference. In Figure 2a, four prominent peaks were observed at 2*θ* = 22.8°, 32.5°, 46.6°, and 57.9°, corresponding to the (001), (110), (002), and (112) diffraction planes of the BNBST films, respectively. Additionally, a diffraction peak attributed to the Pt (111) plane was identified at 2*θ* = 39.9°. A sharp peak near the (110) peaks at 2*θ* = 33.0° for x = 0.02 and 0.11 was attributed to the Si (002) of the substrate. The absence of secondary phase peaks in the XRD patterns confirmed a pure perovskite structure of all the BNBST films and the forming of a uniform and stable solid solution with varying Ba^2+^ ions content.

The XRD spectra further revealed a polycrystalline structure for all films with no evidence of preferential orientation. To gain deeper insights into the film structure, a slow scan of the films in the 2*θ* range of 45° to 48° was presented in Figure 2b. The magnified (002) peaks for all films were fitted well using a single peak, which broadened but did not exhibit any noticeable splitting, suggesting that the films maintain a pseudocubic structure. It was reported that BNBST ceramics exhibited pseudocubic symmetry with x < 0.08, and the tetragonal phase arose with the splitting of (002) peaks when x > 0.08 [33]. The pseudocubic structure in the films with x ranging from 0.02 to 0.11 may be due to the substrate clamping effect and the fine grains, similar to the results in other BNT-based films [35,36]. The increase of Ba^2+^ (r = 1.35 Å) content gradually replaced the original Na^+^ (r = 0.99 Å) and Bi^3+^ (r = 0.96 Å) ions at the A-site, leading to lattice expansion due to the larger ionic radius of Ba^2+^ [37]. This expansion caused a shift of the XRD peaks toward lower 2*θ* values, as demonstrated in Figure 2b. The peak profiles of the films in Figure 2a were refined using a cubic form by Jade 6.5 software. The lattice parameters were determined to be 3.905 nm, 3.911 nm, and 3.021 nm for films, with x = 0.02, 0.06, and 0.11, respectively. The Ba^2+^ ionic got into the lattice located at the A-site, increasing the disorder of the A-site occupation and the unit cell dimension.

To further investigate the peak broadening effect of the films, the Williamson-Hall (W-H) method is an easy way to analyze the grain size and micro-strain contributions. The modified W-H method, using the uniform deformation model equation [38], as presented:

(3) $$\beta_{hkl}\cdot cos\theta =\frac{k\lambda }{D}+4\varepsilon \cdot sin\theta$$

where *λ* is the incident radiation, *D* is the average grain size, *k* is a constant coefficient, *θ* is the diffraction angle, *β* is the FWHM, and *ε* is the micro-strain. Figure 2c showed the plotting of Equation (3), where 4sin*θ* was the *X*-axis and the β_hkl_·cos*θ* was the *Y*-axis. The data was linear fitted well, where the correlation coefficient values of R^2^ were lower than 1 for all the films. The average grain size can be calculated from the intercept of the fitted lines, which were 55 nm (x = 0.02), 60 nm (x = 0.06), and 53 nm (x = 0.11). The slope refers to the intrinsic strain, and the value monotonically increased with the Ba^2+^ of the films, increasing from 0.15% (x = 0.02) to 0.20% (x = 0.11). The position slope values confirmed the lattice expansion [39], and more Ba^2+^ ionic induced an increasing lattice strain in the films.

**Figure 3:**
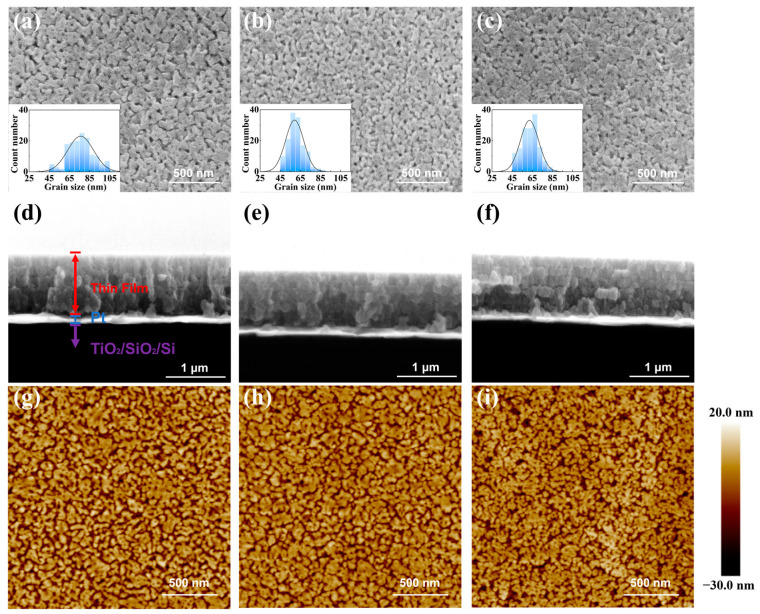
Morphology images of (Bi_0.5_Na_0.5_)_0.8−x_Ba_x_Sr_0.2_TiO_3_ films: (**a**–**c**) surface SEM images and corresponding grain size statistics of the film when x = 0.02, 0.06, and 0.11, respectively; (**d**–**f**) sectional SEM images of films: (**d**) x = 0.02, (**e**) 0.06, and (**f**) 0.11; AFM images of films: (**g**) x = 0.02, (**h**) 0.06, and (**i**) 0.11.

Figure 3a–c displayed the surface morphologies of the BNBST films with different Ba^2+^ content (x = 0.02, 0.06, 0.11) with no visible cracks. The grain size distributions, as inset in Figure 3a–c, indicated that the mean grain size in film with x = 0.06 (59 nm) and 0.11 (62 nm) was smaller than that in x = 0.02 (76 nm). The surface morphologies exhibit lateral grain size, and the Williamson-Hall analysis provides grain size in the film’s normal direction. The grain sizes measured from the morphologies were in accordance with that by XRD analysis, indicating the grain is spherical in shape with a diameter around sub-hector nanometers. Although the grain size is largely smaller in films (tens of nanometers) than in ceramics (a few micrometers), the variation of the grain with Ba^2+^ content is similar to that in ceramics [40]. The grain in BNBST film with x = 0.06 was uniform and looked denser than x = 0.11. This is because larger Ba^2+^ ionic increases the lattice size and induces more strain in the films, resulting in more pores between grain boundaries to release stress. The sectional SEM images of the BNBST films deposited on the Pt/TiO_2_/SiO_2_/Si substrates were shown in Figure 3d–f. The sectional SEM images showed that the thickness of the BNBST (x = 0.02, 0.06, 0.11) films was in the range 0.9~1 μm. The films exhibited a dense columnar structure oriented normally to the surface, with tightly connected grains between the layers. The Root mean square roughness (Rq) determined from the AFM images in Figure 3g–i decreased from 6.83 nm (x = 0.02) to 6.13 nm (x = 0.06) and then increased to 6.42 nm (x = 0.11). Although porosity was observed in the film surface, the cavities did not penetrate in the thickness direction. The films performed low loss and were sufficient for achieving excellent electrical performance.

**Figure 4:**
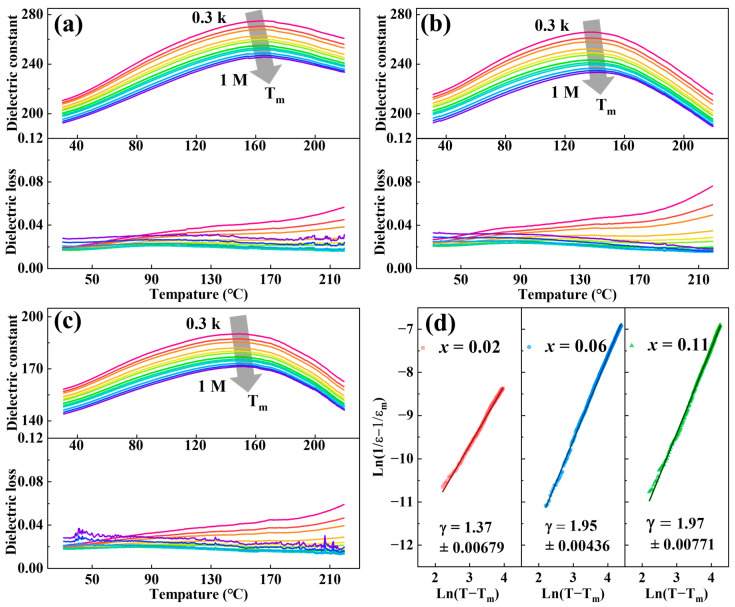
Dielectric temperature spectrum of (Bi_0.5_Na_0.5_)_0.8−x_Ba_x_Sr_0.2_TiO_3_ films: (**a**) x = 0.02; (**b**) x = 0.06; and (**c**) x = 0.11. (**d**) The fitted curve according to the modified Curie–Weiss law.

Figure 4 presented the temperature-dependent dielectric constant (*ε*_r_) and dielectric loss of BNBST (x = 0.02, 0.06, 0.11) films measured over a temperature range from 30 °C to 220 °C and a frequency range from 300 Hz to 1 MHz. The dielectric constant and loss as functions of temperature were shown in Figure 4a–c. The dielectric constant of all films exhibited a frequency-dependent decrease, indicating a typical dielectric dispersion behavior. A dielectric anomaly was observed at the temperature *T*_m_, where the dielectric constant reached its maximum value (*ε*_rm_) [33,41]. The *T*_m_ slightly shifted to a higher temperature with an increase in frequency, which is a feature of relaxor behavior. This broad *ε*_rm_ peak characterizes the typical diffuse phase transition from the relaxor ferroelectric phase to the paraelectric phase [42]. All the films exhibited low dielectric loss (<0.08) and faintly varied with Ba^2+^ content, which is indicative of the high quality and unpenetrated pores of the prepared films. The dielectric loss, for example, at 1 kHz and 30 °C (near room temperature), is lower than 0.03, which is significantly lower than that in BNTST ceramics (0.05) [33] and comparable with that in BNT-based thin and thick films [36,43]. The low dielectric loss is essential for practical applications, as it ensures minimal energy dissipation during device operation. To further explore the relaxor behavior of the films, the modified Curie–Weiss law was applied to evaluate the degree of relaxation [44,45,46]:

(4) $$\frac{1}{\varepsilon_{r}}-\frac{1}{\varepsilon_{\mathrm{rm}}}=\frac{{\left(T-T_{m}\right)}^{\gamma }}{C},\;T>T_{m}$$

where *ε*_r_ is the relative dielectric constant at temperature *T*, and *C* is a constant similar to the Curie constant. The coefficient *γ* reflects the degree of ferroelectricity or relaxation in the material, with a value close to 1 indicating ideal normal ferroelectricity and a value close to 2 indicating classic relaxor behavior. The fitting results of the data from Figure 4a–c were shown in Figure 4d. These results confirm the relaxor nature of the BNBST films. The relaxor degree was enhanced by the increase in Ba^2+^ content. The film with x = 0.02 was between the normal ferroelectricity and ideal relaxor state with *γ* = 1.37. In contrast, films with Ba^2+^ content exceeding 0.06 showed *γ* values approaching 2, indicating that they are close to the ideal relaxor ferroelectric state. The diffuseness behavior in films is consistent with that in BNBST ceramics, in which the *γ* value reported increases from 1.59 to 2 for 0.02 ≤ x ≤ 0.1 [33]. This composition-dependent diffuseness behavior is related to the increase in the degree of lattice disorder by introducing Ba^2+^ at the A-site [47].

**Figure 5:**
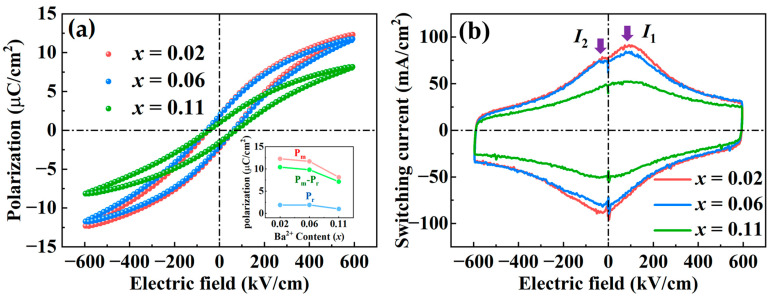
(**a**) Polarization loops and (**b**) corresponding switching current loops of the (Bi_0.5_Na_0.5_)_0.8−x_Ba_x_Sr_0.2_TiO_3_ (x = 0.02, 0.06, 0.11) films under an electric field of 600 kV/cm at 1 kHz. The *P*_m_, *P*_r_, and *P*_m_-*P*_r_ values as a function of the Ba^2+^ content are presented as an inset in (**a**).

Figure 5 illustrated the polarization (*P*-*E*) loops and corresponding switching current (*J*-*E*) loops of the BNBST films under an applied electric field of 600 kV/cm at a frequency of 1 kHz. In Figure 5a, the *P*-*E* loops of all compositions show an elongated shape with low remnant polarization, which is a typical relaxation behavior [48,49,50]. Compared with ceramics, the films with low Ba^2+^ content exhibited slim *P*-*E* loops due to the clamping effect from the substrate. The clamping effect also leads to a larger coercive electric field in films than in ceramics and reduces the ferroelectric variation between different film compositions. The maximum polarization (*P*_m_) and spontaneous polarization (*P*_s_) decreased with the increase of Ba^2+^ content in the films. This increasing relaxor behavior with film composition is in conformity with the evolution behavior of the dielectric temperature spectrum in Figure 4 and can be further demonstrated by the *J*-*E* loops, as shown in Figure 5b. The switching current loops for BNBST films with x = 0.02 and x = 0.06 displayed a prominent peak (*I*_1_) at the coercive electric field, characteristic of ferroelectric polarization switching [51], along with an additional smaller peak (*I*_2_) near the zero electric field. These are essentially the same results observed in the BNT-based materials located at the phase boundary where nonergodic relaxation and ergodic relaxation coexist [52]. The electric field induces a phase transition from ergodic relaxor to ferroelectric transition, which is a reversible process. The current peak, *I*_2_, comes from the recovery of the ergodic relaxor state from the induced ferroelectric phase during the cyclic electric field process. Specifically, the x = 0.02 composition could be closer to the nonergodic side, where active polar nanoregions (PNRS) begin to freeze, leading to a more stable polarization but still showing some relaxor behavior. The x = 0.06 composition, on the other hand, might be closer to the ergodic side, where PNRs are more dynamic and contribute more actively to the polarization under an electric field. This boundary condition enhances both the polarization and the switching currents, as PNRs can still reorient to some extent. In contrast, the film with x = 0.11 exhibited a broadened and flattened current curve, which is typical in an ideal ergodic relaxation state. In this state, the PNRs are highly dynamic, but their random orientation and continuous reorganization under an electric field reduce the overall net polarization and switching currents. The highly dynamic nature of the PNRs in this deep ergodic state disrupts the formation of a stable, strong polarization, leading to the observed lower polarization intensity and weaker switching currents [53].

**Figure 6:**
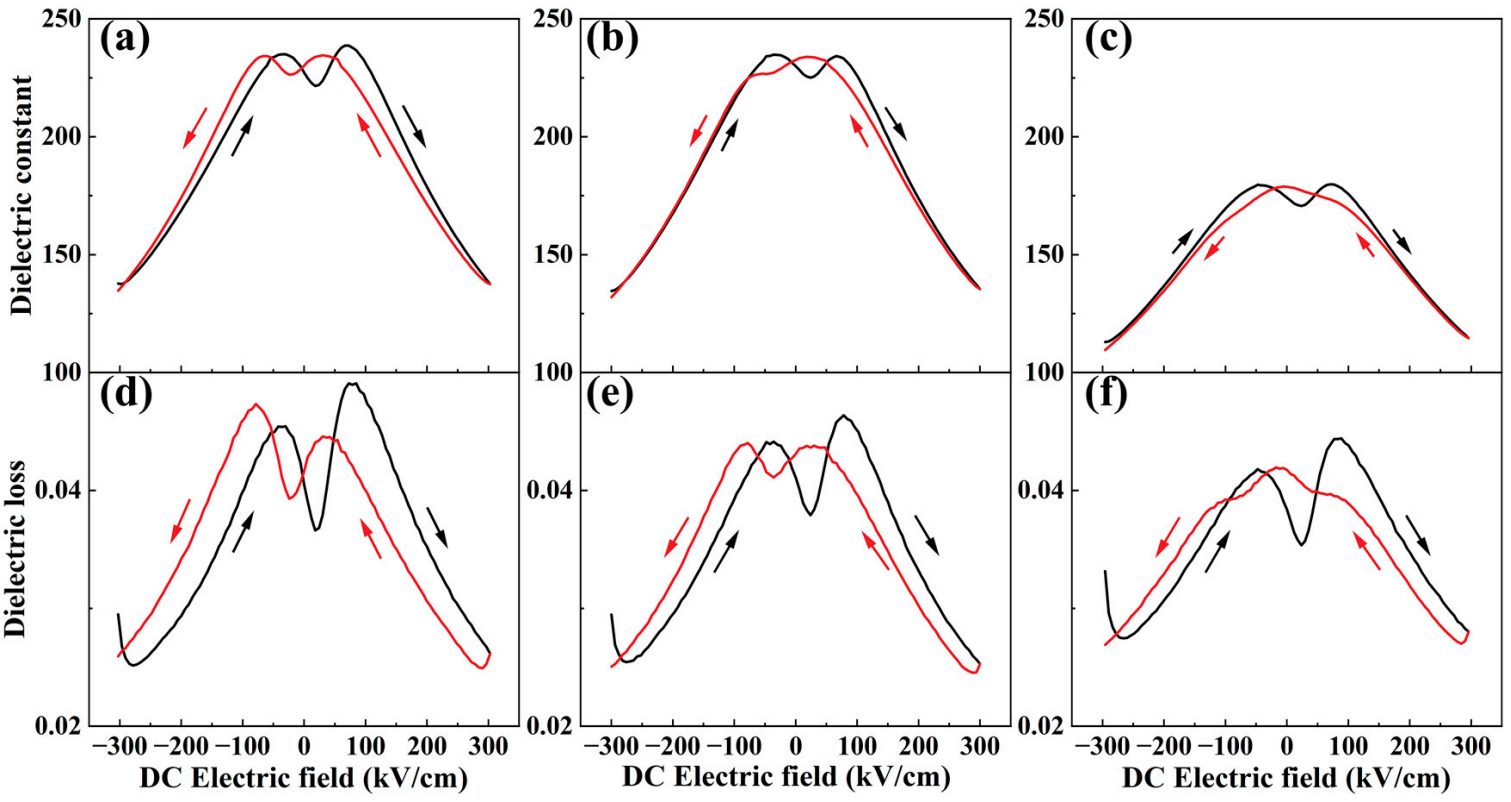
Dielectric constant: (**a**) x = 0.02; (**b**) x = 0.06; (**c**) x = 0.11,and dielectric loss: (**d**) x = 0.02; (**e**) x = 0.06; (**f**) x = 0.11 curves as a function of DC electric field of (Bi_0.5_Na_0.5_)_0.8−x_Ba_x_Sr_0.2_TiO_3_ films at room temperature and 1 kHz.

Figure 6a–f showed the dielectric constant (*ε*_r_-*E*) and dielectric loss (tanδ-*E*) curves of BNBST films at room temperature and 1 kHz under a DC electric field ranging from −300 kV/cm to 300 kV/cm, then back from 300 kV/cm to −300 kV/cm. At a zero electric field, the dielectric constants of the x = 0.02 and x = 0.06 films were similar and higher than that of the x = 0.11 film. All films exhibited low dielectric loss, profiting from their high quality. For the x = 0.02, x = 0.06, and x = 0.11 films, the *ε*_r_-*E* and tanδ-*E* curves show distinct double peaks in one direction, which is a typical feature of films with ergodic relaxor phases [54]. Additionally, the change in the dielectric constant during the applied electric field was much smaller in the x = 0.11 film compared to the x = 0.02 and x = 0.06 films, which is consistent with observations of deeply ergodic BNT-based relaxors reported by Robert et al. [55]. BNBST films exhibit significantly different dielectric response and loss characteristics, depending on their composition. These variations are closely related to the Ba^2+^ content in the films, demonstrating the potential to tune the dielectric properties of the films through compositional adjustments.

**Figure 7:**
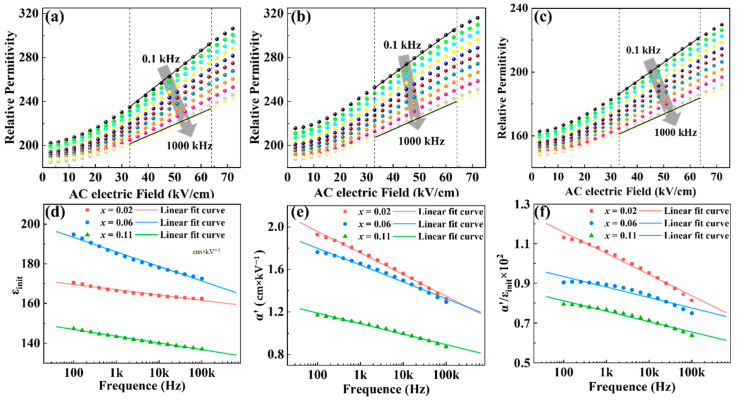
Dielectric constant (*ε*_r_) vs. AC electric field (*E*) measured at various frequencies for (Bi_0.5_Na_0.5_)_0.8−x_Ba_x_Sr_0.2_TiO_3_ films: (**a**) x = 0.02; (**b**) x = 0.06; and (**c**) x = 0.11. Results of the Rayleigh analysis: (**d**) initial dielectric constant (*ε*_init_); (**e**) irreversible Rayleigh coefficient (*α*’); and (**f**) ratio of irreversible Rayleigh coefficient to the initial dielectric constant (*α*’/*ε*_init_).

Using the Rayleigh law, we further analyzed the effect of increasing the Ba^2+^ content on the dielectric properties of (Bi_0.5_Na_0.5_)_0.8−x_Ba_x_Sr_0.2_TiO_3_ (x = 0.02, 0.06, 0.11) films. Figure 7a–c presented the dielectric constant as a function of the driving AC electric field measured at frequencies ranging from 100 Hz to 1 MHz. In the Rayleigh region, where the electric field is less than 1/2*E*_c_, the dielectric constant increased linearly with the amplitude of the driving AC electric field. The AC electric field-dependent dielectric constant *ε*_r_ can be described by the Rayleigh equation as follows [56,57]:

(5) $$\varepsilon_{r}=\varepsilon_{\mathrm{init}}+\alpha^{\prime }E$$

where *ε*_init_ reflects the contributions from lattice distortion and reversible domain wall motion, while *α*’ is the irreversible Rayleigh coefficient, indicating the contributions from irreversible domain wall motion and phase transitions [58]; *E* is the amplitude of the driving AC electric field.

The Rayleigh coefficients *ε*_init_ and *α*’ were calculated using Equation (5) by fitting the linear region in Figure 7a–c. Figure 7d showed the variation of reversible Rayleigh coefficient *ε*_init_ with frequency for the three films. The film with x = 0.06 exhibited the highest *ε*_init_ values across all frequencies, indicating substantial reversible domain wall motion and lattice distortion contributions. In contrast, Figure 7e demonstrated the irreversible Rayleigh coefficients *α*’ decreased with the increase of Ba^2+^ content. Both the *ε*_init_ and *α*’ decreased when the frequency increased, which is related to the weakened domain wall motion with the frequency increase. The ratio of *α*’/*ε*_init_ was shown in Figure 7f, which exhibited a monotonic decreasing tendency with the variation of film composition. The Rayleigh analysis confirmed the most significant reversible domain wall motion contribution in film with x = 0.06. The irreversible domain wall motion contribution to the dielectric properties significantly decreased with the increase of Ba^2+^ content, which is related to the increase of ergodic relaxor degree in films. High Ba^2+^ content likely disrupts long-range ferroelectric order, reducing domain wall mobility and dielectric performance.

**Figure 8:**
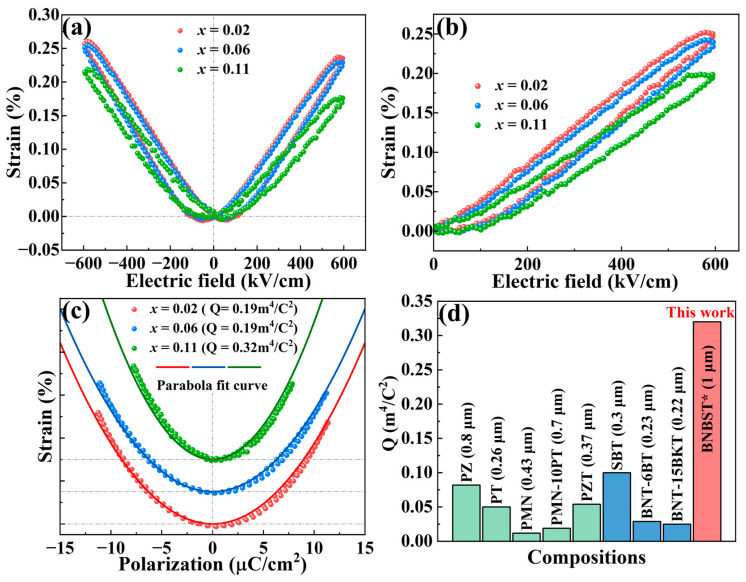
(**a**) The bipolar strain–electric field (*S*–*E*) loops; (**b**) the unipolar strain–electric field (*S*–*E*) loops; and (**c**) the strain–polarization (*S*–*P*) loops measured at 600 kV/cm at room temperature of (Bi_0.5_Na_0.5_)_0.8−x_Ba_x_Sr_0.2_TiO_3_ (x = 0.02, 0.06, 0.11) films. The dotted symbols are experimental data, and the lines fit the data with a parabola in (**c**). (**d**) Comparison of the electrostrictive coefficient of this work with other electrostrictive films reported in the literature [14,15,17,18,26,27,28,59], all the them were prepared by a sol-gel process, BNBST* refers to (Bi_0.5_Na_0.5_)_0.69_Ba_0.11_Sr_0.2_TiO_3_ films.

Strain response measurements were conducted using a ferroelectric analyzer equipped with a double-beam interferometric system. The bipolar and unipolar strain curves of BNBST films under an applied electric field of 600 kV/cm were shown in Figure 8a and b, respectively. Both the bipolar and unipolar *S*-*E* curves exhibited low hysteresis and remarkable strain values higher than 0.2%. The strain values of the x = 0.02 and x = 0.06 films were notably similar, larger than that of x = 0.11, and consistent with their polarization and dielectric constant. This similarity is intriguing and reinforces our earlier conclusions that films with x = 0.02 and x = 0.06 are near the phase boundary between ergodic and nonergodic relaxor ferroelectric states, where an electric field induces a phase transition from ergodic relaxor to ferroelectric state, contributing to the large strain response in these two films.

The *S*-*P* curves, derived from the bipolar strain curves and the simultaneously measured *P*-*E* loops, were well-fitted by parabolic equations in Figure 8c, indicating the predominant contribution from electrostriction. The electrostrictive coefficients (*Q*) were calculated from the fitted results using Equation (2) [25]. The x = 0.11 film demonstrated the highest electrostrictive coefficient, corresponding to the lowest dielectric constant and polarization. The correlative larger strain and larger polarization, as well as larger dielectric constant in films with x = 0.02 and x = 0.06, result in a lower *Q* than x = 0.11. Compared with the electrostrictive coefficients of other films reported in the literature, as presented in Figure 8d, the BNBST films in this work exhibited significantly higher coefficients [14,15,17,18,26,27,28,59]. The ultrahigh electrostrictive coefficient in BNBST thick films is primarily attributed to the high strain response combined with the extremely low polarization value in these compositions, especially in films of x = 0.11, the ergodic relaxor films.

The electrostrictive phenomenon arises from the extension or compression of the crystal lattice by applying an electric field, which is related to the crystal structure and the ionic bond. According to ab initio computation results, BaTiO_3_ exhibited higher ionic bond length and larger *Q* than those in SrTiO_3_ [60]. In our BNBST thick films in this work, more Ba^2+^ content means a higher ratio of BaTiO_3_ in the solid solutions, contributing to an increase in the ionic bond in the lattice, conducive to improving the electrostrictive effect. In addition, the relaxor behavior of BNBST films is significantly influenced by the Ba^2+^ content. When the Ba^2+^ content, x, increased from 0.02 to 0.11, the pseudocubic unit cell sustainably increased, a transformation from the coexistence of nonergodic and ergodic relaxor phases (x = 0.02 and x = 0.06) to ergodic relaxor phase (x = 0.11) occurred, along with the polarization and strain response degeneration in x = 0.11 films. The strain response primarily comes from the electrostrictive effect in x = 0.11 films with an ergodic relaxor state, in which the highest *Q* was obtained. The higher strain response in x = 0.02 and x = 0.06 films originates from the comprehensive result of the field-induced phase transition and electrostrictive effect. These findings highlight the tunability of dielectric and relaxor properties through compositional modifications in BNBST films. The remarkable strain response and electrostrictive behavior are crucial for optimizing their performance in various applications.

## 4. Conclusions

In conclusion, the systematic investigation of (Bi_0.5_Na_0.5_)_0.8−x_Ba_x_Sr_0.2_TiO_3_ (x = 0.02, 0.06, 0.11) thick films, prepared via the sol-gel method on a Pt/TiO_2_/SiO_2_/Si substrate, has yielded promising results. All BNBST films exhibited a pure perovskite structure with a polycrystalline nature and uniform grain sizes with tens of nanometers. The Ba^2+^ located at A-site increased the lattice parameter of films from 3.905 nm (x = 0.02) to 3.021 nm (x = 0.11). The dielectric properties showed typical relaxor behavior, with a diffuse phase transition and the degree of relaxation increased with Ba^2+^ content from *γ* = 1.37 to 1.97. An ultrahigh electrostrictive coefficient *Q* of 0.32 m^4^/C^2^ was achieved in the x = 0.11 films, benefiting from its remarkable strain response (*S* = 0.2%) and fairly low dielectric constant (155) and polarization (*P*_max_ = 8 μC/cm^2^). This makes BNBST lead-free film a promising candidate material for advanced electrostrictive actuator integration into next-generation microelectromechanical systems (MEMS). It also provides suggestions for research into other electrostrictive films, i.e., by taking into consideration high strain and low dielectric constant in a solid solution with an ergodic relaxor state.

## Data Availability

Data is contained within the article.
